# Effectiveness and safety of VISULAS® green selective laser trabeculoplasty: 12 months retrospective data

**DOI:** 10.1007/s10103-024-04252-3

**Published:** 2024-12-15

**Authors:** Karin R. Pillunat, Florian T. A. Kretz, Stefan Koinzer, Philipp Müller, Lutz E. Pillunat, Karsten Klabe

**Affiliations:** 1 Department of Ophthalmology, Medical Faculty Carl Gustav Carus, Technical University, Fetscherstrasse 74, 01307 Dresden, Germany; 2 Precise Vision Augenärzte, Augentagesklinik Rheine, Osnabrücker Str. 233-235, 48429 Rheine, Germany; 3 Augenarztpraxis Am Dreiecksplatz / Kiel, Holtenauer Straße 1, 24103 Kiel, Germany; 4 Internationale Innovative Ophthalmochirurgie GbR, Martin-Luther-Platz 22/26, 40212 Düsseldorf, Germany

**Keywords:** Selective laser trabeculoplasty, SLT, Open-angle glaucoma, POAG, VISULAS green laser

## Abstract

To report the safety and effectiveness of selective laser trabeculoplasty (SLT) using the SLT mode of the VISULAS^®^ green laser in patients with primary open-angle glaucoma (POAG). Twelve months results are presented. Retrospective extension in 4 German centers of an initially prospective interventional multicenter 3-month clinical investigation using the VISULAS^®^ green SLT (Carl Zeiss Meditec AG, Jena, Germany) in patients with POAG who either needed treatment escalation or commenced treatment and had an IOP ≥ 17mmHg at baseline, with no previous glaucoma or other ocular surgery. Non-overlapping laser spots (100) were applied in a single session to 360° of the trabecular meshwork (TM). Glaucoma medications were not changed up to the 3-month visit. From 3 to 12 months, patients were managed according to routine standard of care. Outcome measures included IOP reduction, further glaucoma interventions, and adverse events from baseline to month 12. 25 eyes of 25 POAG patients (mean age 65.8 ± 8.5; modified intention to treat – mITT –group) were included in the extension study. Six eyes (24%) underwent additional glaucoma treatment or changed glaucoma therapy; the remaining 19 eyes (76%) had stable glaucoma therapy (SGT group) with no further glaucoma intervention or change in glaucoma medications (mean number of preoperative glaucoma medications: 2.3 ± 1.34). In the SGT group, mean baseline IOP (mmHg) was reduced from 20.0 ± 2.11 at baseline to 17.4 ± 3.25 and 16.2 ± 1.83 at 6 to 12 months, respectively (*p* < 0.0001): 52.6% had ≥ 20% IOP reduction at 12 months. Potential device- or procedure-related adverse events were mild to moderate and resolved without sequelae. SLT performed with the VISULAS^®^ green laser reduced IOP in eyes with POAG up to 12 months with no relevant safety issues. The results are comparable to other reported SLT studies.

## Introduction

Glaucoma is a complex eye disorder characterized by damage to the optic nerve head, and the main treatable risk factor of this damage is usually increased intraocular pressure (IOP). The mainstay of evidence-based treatment for all types of glaucoma is currently the reduction of IOP [1–3]. The primary goal in managing this vision-threatening optic nerve condition, which results from the damage and apoptosis of retinal ganglion cells, is to preserve visual function and prevent severe visual field defects.

In the last twenty years, selective laser trabeculoplasty (SLT) has emerged as a well-tolerated and increasingly important method to lower IOP in patients with primary open-angle glaucoma (POAG). It has even been hailed as a promising “new star” in glaucoma treatment [4]. Part of its growing appeal lies in its safety profile combined with its ability to reduce dependence on topically applied anti-glaucoma medications, which are typically the first-line treatment but often face challenges due to non-adherence or intolerance.

SLT is typically performed using a frequency-doubled Q-switched 532 nm Nd:YAG laser. Instead, the VISULAS^®^ green laser is a frequency-doubled diode-pumped 532nm Nd:YVO_4_ laser, which enables the instrument to be an integrated retina and glaucoma laser that can operate in a selective mode to perform SLT. Unlike conventional SLT lasers, no cavitation bubbles are visible during treatment, and the initial laser energy is determined by the Scheie degree of pigmentation. This approach eliminates the need for the titration process required by traditional SLT lasers, thereby reducing treatment time and minimizing unnecessary energy exposure to the eye. Additionally, VISULAS green laser differs from the conventional SLT in that it utilizes a 400 μm laser spot formed by the sequential application of 52 square spots, each with an edge length of 50 μm. This results in a more uniform distribution of laser energy. The fluence, a crucial factor in treatment effectiveness, is comparable to that of conventional SLT lasers [5]. The laser acts by selective photothermolysis targeting the pigmented trabecular meshwork while sparing non-pigmented structures, ensuring no lasting tissue damage occurs [6]. Although SLT has proven effective, the exact mechanisms responsible for its ability to reduce (IOP) and enhance trabecular outflow are not yet entirely understood. Yet, some propose that the biological and repopulation theories provide a sufficient explanation for how SLT achieves its IOP-lowering effect [7, 8].

The effectiveness and safety of SLT have been demonstrated as primary[9–11] as well as adjunctive therapy [12, 13]. The primary objective of the current study was to assess the effectiveness and safety of this new SLT technique using the VISULAS^®^ green laser (Carl Zeiss Meditec AG, Jena, Germany) in patients diagnosed with POAG. To our knowledge, this is the first study to evaluate the long-term effectiveness and safety of SLT using the VISULAS^®^ green laser.

## Methods

This is a retrospective, observational extension of a 3-month prospective interventional multicenter clinical investigation which was performed in 4 research centers across Germany: Department of Ophthalmology, Universitätsklinikum Carl Gustav Carus, Dresden; Precise Vision, Rheine; Augenarztpraxis am Dreiecksplatz, Kiel; Breyer, Kaymak & Klabe Augenchirurgie, Düsseldorf. Patients who needed either treatment escalation or commenced treatment and had an IOP ≥ 17mmHg at baseline with no previous glaucoma or other ocular surgery were recruited in the original study.

The prospective 3-month results are published in the literature [5]. The present study evaluated the same cohort of patients with POAG on the 12-month efficacy and safety profile of VISULAS^®^ green SLT. Eligibility and criteria, as well as data collection, parameters, and treatment with VISULAS^®^ green laser were previously reported [5].

Twenty-five patients were included; from 3 to 12 months, patients were managed according to routine standard of care. After 3 months, the clinical protocol was no longer enforced in all patients, so this study is now considered retrospective and observational.

Approximately one hundred (100) non-overlapping laser spots were applied in a single session to 360° of the trabecular meshwork (TM), and the energy was adjusted according to the pigmentation of the TM. Glaucoma medications were not changed up to the 3-month visit. Participants of the initial 3-month study who were willing and able to return for follow-ups at 6 and 12 months were included.

### Groups

All eyes enrolled were assigned to the modified intention to treat (mITT) group. The eyes that did not undergo additional glaucoma surgery or change in medical therapy were assigned to the stable glaucoma therapy (SGT) group. The results of this manuscript will focus on the SGT group, and we will report on the mITT group about the safety profile.

### Endpoints

All endpoints were exploratory only. Outcomes included IOP reduction, percentage of patients with 20% IOP reduction, and change in visual field at 12 months. Time required for secondary glaucoma intervention, as well as adverse events, were also recorded from baseline to month 12.

### Statistical analysis

An independent statistician performed statistical analyses. Sample size estimation was performed with PASS 15 (PASS 15 Power Analysis and Sample Size Software (2017). NCSS, LLC. Kaysville, Utah, USA, ncss.com/software/pass). Demographics and laser parameters were reported using mean, standard deviation, minimum, and maximum. Changes from baseline were analyzed using an ANCOVA model with baseline as a cofactor. Analyses were performed using SAS 9.4 (SAS Institute, Cary, NC, USA). Plots were generated using R [5] version 3.3.3. A p-value < 0.05 was considered statistically significant.

## Results

25 eyes of 25 POAG patients underwent treatment using SLT mode of VISULAS^®^ green laser and were followed up for a period of 12 months to evaluate the long-term efficacy and safety profile. All patients included in the study were of white/European origin. Demographics and baseline characteristics are summarized in Table 1.

**Table 1:** Demographics and baseline parameters

Patients	N (%); mean ± SD
Modified intent-to-treat (mITT) population	25
Age (years)	65.8 ± 8.50
Gender	
* Male*	13 (52.0%)
* Female*	12 (48.0%)
Race	
* White*	25 (100%)
Stable glaucoma therapy (SGT) population	
n	19
Age	65.3 ± 8.01
Gonioscopy (angle width)	
* Shaffer 3 – 20° to 35°*	12 (63.2%)
* Shaffer 4 – 35° to 45°*	7 (36.8%)
Scoring of pigmentation	
*None*	1 (5.3%)
* I – Just visible*	4 (21.1%)
* II – Mild*	6 (31.6%)
* III – Marked*	8 (42.1%)
* IV – Intense*	0 (0%)
IOP (mmHg)	20.0 ± 2.11
Visual Field MD (dB)	−5.0 ± 3.77
Visual Field PSD (dB)	5.6 ± 3.51
Subjective cup/disc ratio	0.6 ± 0.14
Glaucoma medications (no.)	2.2 ± 1.42
Distribution by no. of glaucoma medications	
0 meds	3 (15.8%)
1 med	4 (21.1%)
2 meds	3 (15.8%)
3 meds	5 (26.3%)
4 meds	4 (21.1%)

Five out of these 25 patients needed a secondary glaucoma intervention; 1 patient had a reduction in glaucoma medications. The remaining 19 eyes of 19 patients were included in the analysis as the SGT group (Table 1).

**Fig. 1:**
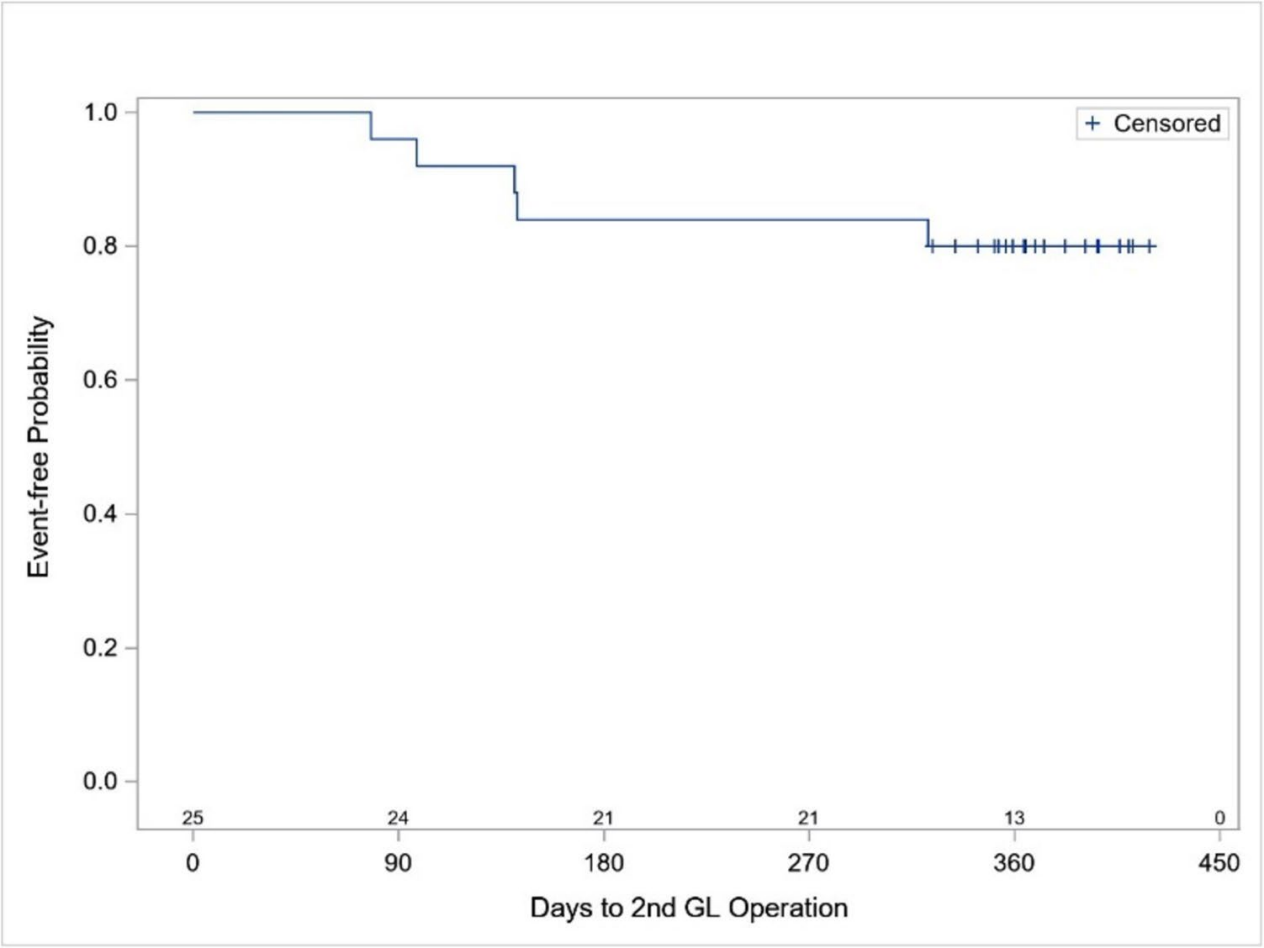
Kaplan–Meier graph of eyes that underwent additional glaucoma surgery or change in glaucoma therapy

The Kaplan–Meier graph (Fig. 1) shows the eyes that underwent additional glaucoma surgery or had to increase the topical glaucoma therapy: 1 eye at 3 months needed additional glaucoma medications, 3 eyes at 4, 5, and 12 months needed an additional SLT procedure (2 eyes received an additional SLT due to medications reduction and 1 eye due to insufficient IOP control) and 1 eye at 5 months was implanted with a trabecular micro-bypass stent (iStent; Glaukos, Burlington, USA) combined with cataract extraction due to insufficient IOP control. In addition, the sixth eye had a reduction in medications and was excluded from the final analysis. Since only 5 of 25 patients (20%) had an event, the median time to events and the corresponding 95% confidence interval were not estimable.

### Stable Glaucoma Therapy (SGT) group - Efficacy analysis

Nineteen eyes (19/25) did not have any change in medications after SLT and were included in the analysis (mean age 65.3 ± 8.01 years). Table 1 shows the distribution of patients according to the number of glaucoma medications, the baseline visual field data, and the distribution of the angle pigmentation degrees as well as the width of the angle according to Shaffer’s classification. Mean number of medications was 2.2 ± 1.42.

### IOP reduction

**Fig. 2:**
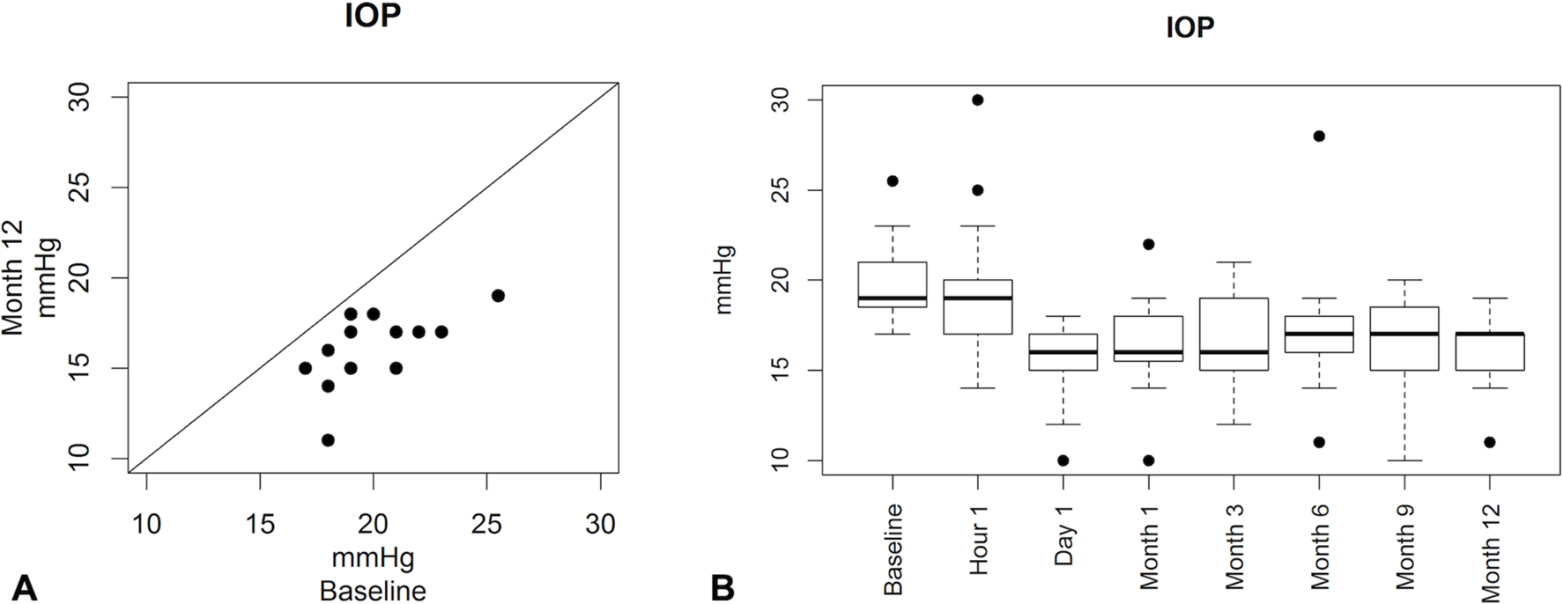
**A** Scatterplot of IOP in SGT group at 12 months postoperatively; **B** box plot of IOP distribution in SGT group at different points in time

In the SGT group, IOP was reduced from a mean baseline value of 20.0 ± 2.11 mmHg to 17.4 ± 3.25 mmHg at 6 months, which was further reduced to 16.2 ± 1.83 mmHg at 12 months (a reduction of 3.82 mmHg; *p* < 0.0001). This translated to a 12.8% reduction from baseline at 6 months and an 18.8% reduction at 12 months (Table 2). Eighteen patients (94.7%) had a ≥ 10% reduction in IOP from baseline to 12 months; 10 (52.6%) patients had a ≥ 20% reduction in IOP. Figure 2 is a scatterplot showing IOP outcomes of the SGT group at 12 months.

**Table 2:** Mean percentage change in IOP from baseline: modified intent to treat (mITT) group and stable glaucoma therapy (SGT) group

	Relative change in IOP (%) *mean* ± *SD*				
	1 M	3 M	6 M	9 M	12 M
mITT group (*n* = 25)	−15.6 ± 15.70	−15.6 ± 14.37	−18.5 ± 17.12	−15.4 ± 14.39 (*n* = 22)	−21.3 ± 10.63
SGT group (*n* = 19)	−16.6 ± 15.97	−15.9 ± 15.35	−12.8 ± 14.30	−15.2 ± 11.67 (*n* = 16)	−18.8 ± 8.26

### Change in visual field

Visual field measurements were only available for a limited number of study eyes in the SGT group (*n* = 7 at 12 months), in which no significant change of visual field parameters from baseline to 12 months was observed. For those 7 eyes, mean deviation (MD) changed from mean −4.3 ± 3.40 dB (range: −10.0 to 0.5) at baseline to −4.0 ± 2.79 dB (range: −6.8 to 0.7) after 12 months. Pattern standard deviation (PSD) of the same eyes was 4.8 ± 2.66 (range: 1.7 to 9.0) at baseline and 5.0 ± 3.15 (range: 2.2 to 10.5) at 12 months.

### Adverse events

In the original cohort of 34 patients in the 3-month prospective interventional multicenter clinical investigation, three patients had a transient (less than a day) increase in IOP directly after the treatment, potentially related to the device or the SLT procedure. Five patients reported eye pain or discomfort that was judged related to the procedure. All these events were transient and resolved without any sequelae [5].

One of the 25 patients enrolled in the extension study underwent cataract surgery over the 12-month period of the study. The patient underwent cataract surgery and iStent implantation due to insufficient IOP control, making the cataract unlikely to be related to the device or the procedure. No serious adverse events, such as uveitis, corneal edema, choroidal effusion, peripheral anterior synechiae, or cystoid macular edema occurred among the study population.

## Discussion

This is a retrospective extension of an initially prospective interventional multicenter 3 months clinical investigation using the VISULAS^®^ green SLT in patients with POAG, who either needed a treatment escalation or commenced treatment and had an IOP ≥ 17mmHg at baseline, with no previous glaucoma or other ocular surgery [5].

At 12 months, 94.7% of patients from the SGT group showed an IOP reduction of ≥ 10% compared to the baseline values. Meanwhile, a ≥ 20% reduction of IOP from baseline was seen in 52.6% of the cases in the SGT group. Adverse events were mild and, in most cases, not directly related to the procedure, and only the commonly noted side effects of SLT were observed: transient IOP spikes, eye pain, or discomforts. These are known side effects of SLT with incidence comparable to the literature [14].

Success of SLT treatment is commonly defined as the proportion of eyes that achieve a reduction in IOP of ≥ 20% from baseline without an increase in glaucoma medication and/or repeat glaucoma laser or surgical procedure [7]. According to this definition, success was achieved in 52.6% of eyes in the SGT group in our study, which is similar to results reported by other SLT studies [15].

Further, our study reports 12.8% IOP reduction from baseline at 6 months and 18.8% reduction at 12 months in the SGT group. These results are similar to other documented SLT studies [16–19]. In a systematic review, Wong et al. [16] found a mean relative IOP reduction of 14.7% from a baseline IOP of 21.3 ± 4.7 mmHg, with 40.3% showing a ≥ 20% IOP reduction 12 months post-SLT. In another 2020 retrospective study, Elahi et al. [17]. documented a 17.6% reduction in IOP from baseline at 12 months, with a 27.4% success rate. In a similar 6-month study by Chadwick et al. [18], a mean relative IOP reduction of 16.7% was found 6 months post-SLT. According to the previous studies, the only identifiable factor influencing IOP post-SLT was the baseline IOP: the higher the baseline IOP, the greater the reduction in IOP observed post-procedure [7, 13, 20]. This was also noted in our study population.

Another noteworthy observation was that SLT does not have a 100% success rate and may not work on all patients. Our study had 5 patients (20%) who had uncontrolled IOP and had to be treated with secondary procedures like routine SLT/iStent/increase in glaucoma medications, and this is aligned with other studies in the literature [13, 15].

The cause for a significant number of eyes not responding sufficiently to SLT remains uncertain. Recent evidence suggests that POAG might impact not only the trabecular meshwork but also structures beyond it, such as Schlemm’s canal and the collector channels. Genetic variations also contribute to this phenomenon. Consequently, if the outflow resistance is primarily caused by issues in Schlemm’s canal or the collector channels, a targeted therapy focused on the trabecular meshwork, like SLT, may not be effective in all cases [21, 22].

The most common ocular adverse events noted during SLT include mild and transient eye discomfort or pain and post-procedure IOP spikes. The eye pain and discomfort were mainly experienced immediately after the procedure and were often associated with the use of the contact lens during the process. Additionally, transient increase in IOP is a frequently reported side effect of SLT and was noted in 3 patients out of 34 of the original cohort (8.8% – incidentally, they are all in the SGT group) in the current study, which is in accordance with the previous studies: Wong et al. found that the incidence of IOP spikes varies between 0% to 28.8% [15]. Similarly, Latina et al. reported that 24% of cases experienced transient IOP spikes of 5 mmHg or more [23].

Notably, none of the cases in this study exhibited severe uveitis, corneal edema with stromal haze, choroidal effusion, hyphema, or peripheral anterior synechiae, nor did they experience retinal complications such as cystoid macular edema or macular burns. Although these complications are rare, they have been reported in some instances following SLT [14, 15].

Limitations of our study include a retrospective design, along with a small sample size. Further, the IOP measurements in the extension study were neither masked nor taken at the same time of the day for each patient, which does not exclude the effect of diurnal variations. Also, this study was conducted exclusively on white/European patients, and most of them had mild to moderate trabecular meshwork pigmentation; therefore, the conclusions drawn from this study may not be generalizable to patients of other ethnicities or those with different degrees of trabecular meshwork pigmentation. Finally, it was focused solely on patients diagnosed with POAG. Further studies with a larger sample size, different ethnicities, and masked IOP measurements can add to our information. An important strength of the present study is the subgroup evaluation of the effect of SLT on a group without any change in glaucoma medication or treatment (stable glaucoma treatment group).

To summarize, the use of the VISULAS^®^ green laser for SLT exhibited substantial effectiveness in lowering IOP in eyes diagnosed with primary open-angle glaucoma. Approximately half of the treated eyes achieved an additional 20% reduction in IOP, establishing clinically significant efficacy. The success rates observed in this study are comparable to those reported in the literature when using other conventional SLT lasers. Furthermore, SLT with the VISULAS^®^ green laser demonstrated a favorable safety profile, aligning with previous reports on the safety of SLT procedures. Overall, these findings indicate that SLT with the VISULAS^®^ green laser can be considered a viable and safe treatment option for managing IOP in patients with POAG.
